# New Technologies for Surgery of the Congenital Cardiac Defect

**DOI:** 10.5041/RMMJ.10119

**Published:** 2013-07-25

**Authors:** David Kalfa, Emile Bacha

**Affiliations:** Pediatric Cardiac Surgery, Columbia University, Morgan Stanley Children’s Hospital of New York-Presbyterian, New York, USA

**Keywords:** Congenital heart defect, extracellular matrix patch, new technologies, percutaneous valve implantation, robotics, tissue engineering

## Abstract

The surgical repair of complex congenital heart defects frequently requires additional tissue in various forms, such as patches, conduits, and valves. These devices often require replacement over a patient’s lifetime because of degeneration, calcification, or lack of growth. The main new technologies in congenital cardiac surgery aim at, on the one hand, avoiding such reoperations and, on the other hand, improving long-term outcomes of devices used to repair or replace diseased structural malformations. These technologies are: 1) new patches: CorMatrix® patches made of decellularized porcine small intestinal submucosa extracellular matrix; 2) new devices: the Melody® valve (for percutaneous pulmonary valve implantation) and tissue-engineered valved conduits (either decellularized scaffolds or polymeric scaffolds); and 3) new emerging fields, such as antenatal corrective cardiac surgery or robotically assisted congenital cardiac surgical procedures. These new technologies for structural malformation surgery are still in their infancy but certainly present great promise for the future. But the translation of these emerging technologies to routine health care and public health policy will also largely depend on economic considerations, value judgments, and political factors.

## INTRODUCTION

Congenital cardiac surgery frequently requires additional tissue such as patches, conduits, and valves. These prostheses are characterized by a risk of degeneration, calcification, and a lack of growth, and usually need a replacement over a patient’s lifetime. The main new technologies in congenital cardiac surgery aim at improving long-term outcomes of these devices and avoiding reoperations.

## NEW PATCHES: CORMATRIX® EXTRACELLULAR MATRIX PATCHES

Despite improvements in congenital heart surgery procedural mortality, there remain a substantial number of patients who need multiple reinterventions,^1^ because of the lack of growth potential and remodeling of currently used patches (autologous pericardium (with or without glutaraldehyde), preserved xenopericardium, and various prosthetic materials). As a matter of fact, the ideal patch still does not exist. Such an ideal material would not interfere with the patient’s growth, would be pliable, soft, resistant to tearing, calcification, and shrinkage, and would possibly not induce remodeling of scar tissue.

Recently, the CorMatrix® (CorMatrix Alpharetta, GA) patch made of decellularized porcine small intestinal submucosa extracellular matrix (SIS-ECM) has been introduced into cardiac surgery. The extracellular matrix (ECM) is the acellular component that surrounds cells in native tissues and is mainly composed of elastin, collagen (structural proteins), glycans (glycosaminoglycans, proteoglycans), and adhesion glycoproteins. These new patches have demonstrated patch remodeling and integration in animal models of cardiac surgery.^2^,^3^ Wainwright et al.^4^ showed that right ventricular outflow tract (RVOT) reconstruction with SIS-ECM patches in a rat model resulted in new cardiac tissue formation in the patched areas and the absence of ventricular dilatation, when compared with Dacron reconstructions of the RVOT.

These promising results in experimental studies have then been confirmed in human studies that specifically evaluated the outcomes of SIS-ECM in congenital heart surgery for cardiac and vascular reconstructions.^5^–^7^ Scholl et al.^5^ demonstrated in one case of an explanted patch used for augmentation of the tricuspid valve that SIS-ECM was replaced by organized collagen and populated with endothelial-like cells four months after the implant. Quarti et al.^6^ showed early encouraging results of these CorMatrix® patches used for vascular repair (pulmonary artery, ascending aorta, aortic arch, and right ventricular outflow tract), but also for valve reconstruction (aortic, tricuspid, mitral, and pulmonary valves) and pericardial closure. Witt et al.^7^ demonstrated that SIS-ECM is suitable for the closure of septal defects. But the use of SIS-ECM for the reconstructions of outflow tracts and great vessels in this study carried a small risk of stenosis, especially in patches that form the majority of the vessel circumference. Moreover these studies had rather a short follow-up. Another potential drawback of CorMatrix® ECM patches is the significant variability of the SIS-ECM biomechanical properties between different lots. Contrary to the Surgisis™ trial assessing the clinical use of SIS-ECM for carotid artery repair following endarteriectomy—a study that displayed an increased risk of aneurysm formation—the CorMatrix® lot did not display such a pejorative evolution even when implanted in high-pressure systems. Nevertheless, the limited numbers of patients in studies dealing with the implantation of CorMatrix® in high-pressure systems prevent their authors from speculating regarding the long-term effectiveness of the CorMatrix® in specific high-pressure locations. Long-term outcomes of these ECM patches depend not only on patch biomechanical properties, patch location, and hemodynamic environment, but also on the patient’s immune response. Badylak et al.^8^ showed that the non-cross-linked SIS-ECM incited an immuno-regulatory and proangiogenic macrophage response (leading to remodeling and repopulation of the patch) instead of an inflammatory, scar-forming response (potentially leading to stenosis).

Porcine SIS-ECM is currently approved by the Food and Drug Administration (FDA) for use in humans. Nevertheless, large studies of the growth potential of the porcine SIS-ECM compared to other biomaterials used in cardiac surgery have not been conducted yet.

To summarize, the CorMatrix® ECM displays a lot of potential advantages over other materials currently used in pediatric cardiac surgery, as follows:

- Easily handled and implantable
- Abundant
- Durable (still controversial)
- Minimal scar formation
- Remodeling of the material (no calcification)
- Growth potential (still controversial)

This new biomaterial seems to provide an interim bioscaffold that enables the patient’s own cells to repopulate and repair damaged tissues, which is of particular interest in patients with congenital heart diseases, for valve repair, and vascular reconstruction. But the long-term performance of the SIS-ECM in congenital cardiac applications still needs to be assessed through longitudinal studies of greater magnitude.

## NEW DEVICES

### Percutaneous Pulmonary Valve Implantation: The Melody® Valve

The right ventricle (RV) to main pulmonary artery (PA) conduits that are used to reconstruct the right ventricular outflow tract in congenital heart diseases are prone to develop valvular incompetence and/or obstruction with time. These pejorative evolutions are associated with exercise intolerance, arrhythmias, and an increased risk of sudden death^9^ and require multiple open-heart surgeries to replace the pulmonary valve.

Percutaneous pulmonary valve implantation was introduced as a new treatment option in patients with dysfunctional conduits.^10^,^11^ This technological breakthrough aims at prolonging the lifespan of RV to PA conduits and thus postponing open-heart surgery. The trans-catheter pulmonary valve (Melody®; Medtronic, Minneapolis, MN) is composed of a bovine jugular venous valve and a balloon-expandable stent made of a platinum-iridium wire.

The current largely accepted indications for the use of a Melody® valve include^12^:

- A significant RVOT obstruction, defined as RV pressures > 2/3 of systolic blood pressure (SBP) with symptoms, or > 3/4 of SBP without symptoms
- A severe pulmonary regurgitation and RV dysfunction or RV dilatation or impaired exercise capacity
- Along with morphological criteria allowing a safe implantation site: RVOT dimensions < 22 × 22 mm and > 14 × 14 mm

The implantation procedure is standardized and safe, with a procedural mortality < 0.2%. The main complication to avoid during the implantation is coronary compression or occlusion, which can be evaluated by a pre-implantation balloon inflation in the RVOT. Other complications during implantation are the dislodgement of the device when implanted in distensible and dilated RVOTs and the risk of homograft rupture.

Valve implantation significantly reduces the gradient across the outflow tract, RV pressures, and the pulmonary regurgitation,^13^ and significantly improves symptoms.

Lurz et al.^13^ demonstrated that during a median follow-up of 28 months freedom from reoperation was 93% (±2), 86% (±3), 84% (±4), and 70% (±13), at 10, 30, 50, and 70 months, respectively.

The main complications of the new generation of this innovative technology are late endocarditis and stent fractures in 20%.^14^ These stent fractures are silent in the majority of cases and are treated in symptomatic patients with RVOT stenosis by a Melody® valve-in-valve implantation.

Pulmonary valve implantation is becoming the standard procedure in the treatment of dysfunctional conduits. It has been accepted by the regulatory agencies for distribution and use in Europe in 2006 and US Food and Drug Administration in 2010.

By prolonging the lifespan of RV–PA surgically placed conduits, this innovative technology has reduced the number of multiple open heart operations in children and young adults with congenital heart disease, and may improve their life expectancy and life quality.

As with all evolving new technologies, new generations of Melody valves were created in order to reduce current limitations and extend the spectrum of potential clinical indications. Improvements brought to the Melody® valve during the last few years of development or currently in progress include:

- Device design improvements
- Delivery system improvements
- Patient selection improvements using three-dimensional echography and MRI
- Dilatation with high-pressure balloon after implantation (to reduce residual gradients)
- Stent-in-stent implantation
- Structural improvements to extend this technology to patients with native, dilated, and distensible RVOT

These principles of percutaneous valve implantation are currently investigated in other off-label clinical settings. For instance, valves developed for trans-catheter replacement of the aortic valve were implanted in the pulmonary position for patients with larger annulus.^15^ A new device allowing the implantation of a pulmonary valve in a RVOT previously repaired with a transannular patch is also currently investigated but not published yet.

### Tissue-Engineered Valved Conduits: Decellularized Scaffolds, Polymer Scaffolds, and *in Situ* Regeneration

The ideal RV–PA conduit for reconstruction of the RVOT still does not exist.

Cryopreserved homografts need a revision surgery in 36% and 90% of cases after 10 and 15 years, respectively.^16^–^18^ Hancock conduits need to be replaced after 10 years in 68% of cases, and 50% of Carpentier–Edwards Perimount® (Edwards Life-sciences, Irvine, CA, USA) valves (bioprosthetic stented valve made of bovine pericardium) implanted in children also have to be replaced after 5 years.^19^ Children younger than 2 years old operated with a Contegra® Medtronic conduit have to undergo a revision surgery in 67% of cases for failure.^20^ The reoperations needed to replace a failing conduit carry a significant risk of mortality (1%–3%) and morbidity: hemorrhagic syndrome, cerebral vascular accident, coronary damage, cardiac rhythm alterations, or infection. These complications translate into prolonged hospitalization and attendant costs. Surgical techniques have improved during the last three decades, but conduit failure and morbidity and mortality still occur (Table 1). Autologous pericardial valved conduits for RVOT reconstruction showed superb properties, but data for long-term follow-up are lacking.^21^

**Table 1:** Current Surgical Valved Conduits to Replace the Right Ventricular Outflow Tract.

**Current Surgical Devices**	**Reoperation Rates**	**Limitations**	**Ref.**
Cryopreserved homografts	6%–58% at 5 years, 36%–90% at 15 years, depending on the diameter, age at surgery, and heart defect	No growth potential Immunogenicity and inflammatory response Calcification Structural degeneration Limited availability	18, 22
Stented heterografts (e.g. Hancock^®^ tube: porcine aortic heart valve in a tube made of Dacron^®^)	19% at 5 years, 68% at 10 years, 95%–100% at 15 years, depending on the diameter, age at surgery, and heart defect	No growth potential Early calcification Structural degeneration Pannus formation Excessive stiffness with anatomic compression/distortion	23
Stentless heterografts (e.g. Contegra^®^ tube: bovine jugular vein)	22%–40% at 5 years, depending on the diameter, age at surgery, and heart defect	No growth potential Immunogenicity and inflammatory response Stenosis of the distal anastomosis Pseudoaneurysm of the proximal anastomosis Severe conduit regurgitation	24, 25
Stentless heterografts (e.g. Shelhigh^®^ tube: porcine pulmonary heart valve in a tube made of bovine pericardium)	48%–67% at 1 year, depending on the diameter, age at surgery, and heart defect	**Intimal peel formation at the distal segment** **No growth potential** **Immunogenicity** and inflammatory response Pseudoaneurysm	26
Mechanical valves	Only in older children and adults	No growth potential Anticoagulant therapy required Thromboembolic complications	27

As a consequence of the limited treatment options and the requirements for repeat surgery in children as they grow, new alternatives were investigated to reconstruct the RVOT. The advanced-therapy medicinal products (ATMPs) derived from the concept of regenerative medicine are presently seen as one of the main routes to reduce the above-mentioned risks, with the exception of organ transplantation.

On the basis of these issues, the search for the ideal material to replace the RVOT started. The *in vitro* creation of autologous and living substitute materials by tissue engineering is based on the essential need for growth potential of materials to be used for surgical correction of congenital cardiac defects.

In the last 15 years, different tissue-engineered materials have been proposed to replace the RVOT. Scaffolds were either decellularized allo- or xenogenic biological valved conduits or bioabsorbable prosthetic materials (poly-4-hydroxybutyrate (P4HB), poly-L-lactide (PCLA), polyglycolic acid (PGA)) designed in unvalved patches,^28^–^32^ non-valved tubes,^33^–^35^ or valved tubes.^36^–^40^

#### Decellularized scaffolds

Dohmen et al. published an account of the first clinical implantation of a tissue-engineered heart valve in 2000^41^: an *in vitro* seeded decellularized pulmonary allograft was implanted during a Ross operation in an adult patient. The 10-year clinical results of these tissue-engineered heart valves of the same group were promising despite a limited number of patients.^42^ Da Costa et al.^43^ demonstrated an excellent hemodynamic behavior and a significant decrease in human leukocyte antigen (HLA) class I and II antigens in decellularized allografts compared with standard allografts. Nevertheless pejorative clinical outcomes of this technology were also reported: Simon et al.^44^ showed that the Synergraft technology failed in four grafts after 2 days and 1 year post-implantation and that no recellularization of the decellularized grafts was seen at up to 1 year of follow-up. In 2010, Da Costa et al.^45^ investigated the outcomes of decellularized aortic homograft implants as an aortic root replacement in 41 patients. No reoperations were performed due to aortic valve dysfunction with a maximal follow-up of 53 months.

#### Polymer scaffolds and in situ regeneration concept

The literature reports that polymer scaffolds were seeded (or not) with different types of autologous cells: endothelial cells, fibroblasts, myofibroblasts derived from peripheral vessels,^28^,^32^–^35^,^36^,^37^,^39^ smooth muscle cells derived from aorta or cardiomyocytes.^29^*In vitro* and *in vivo* studies (goats or adult syngenic rats) of these materials implanted in the RVOT demonstrated the biodegradation of the material,^28^,^29^ the endothelialization of the surface of the material,^30^,^37^,^38^ the synthesis of an extracellular matrix,^28^,^33^,^35^,^37^,^38^,^46^ the absence of thrombus or stenosis,^36^ and a low risk of calcification. In 2006, Hoerstrup et al. proved, in a pioneering work, the growth potential of a bioabsorbable non-valved tube seeded with endothelial cells and fibroblasts implanted on the pulmonary artery in a growing lamb model during 100 weeks.^47^ Concomitantly to this biological progress, other synthetic polymers (poly-L-lactic acid (PLLA),^48^ poly(epsilon-caprolactone) (PCL),^49^ poly(styrene-block-isobutylene-block-styrene) (SIBS),^50^ poly(glycerol-sebacate) (PGS)^51^), and other biological materials (fibrin,^52^ collagen,^53^ 3D cardiac extracellular matrix,^54^ or hybrid materials^55^,^56^) were investigated to create tissue-engineered scaffolds for heart valves. Some polymeric matrices were made “bioactive” through the implantation of growth factors on their surface (transforming growth factor beta, bone morphogenetic protein, and vascular endothelial growth factor).^57^,^58^ Other research groups investigated strategies of “homing” and immobilization of circulating host-derived cells.^59^

Materials designed for RVOT reconstruction by tissue engineering using stem cells were first evaluated *in vitro*.^60^ They were bioabsorbable non-valved patches or valved tubes (PGA+/- P4HB or PGA+PLLA). The first stem cells used were human bone-marrow cells that displayed a myofibroblastic differentiation and synthetized an extracellular matrix.^61^ In 2007, autologous peripheral blood-derived endothelial progenitor cells and autologous bone-marrow-derived marrow stromal cells (MSC) were seeded on a bioabsorbable non-valved patch on the pulmonary artery of seven goats with a follow-up of 6 weeks.^62^–^66^ This study showed the development of a living and organized tissue, integrated to the native pulmonary artery. The use of bioreactors for cell culture and maturation in dynamic conditions allowed for the maturation of the tissue-engineered device, the *in vitro* cell differentiation, and the formation of the extracellular matrix.^67^–^72^ A non-invasive percutaneous method of implantation of tissue-engineered heart valves was described by Dr Hoerstrup’s group^73^ and by Emmert et al.^74^ From 2002, the cells used have been derived from human umbilical cord blood, Wharton’s jelly, amniotic liquid, chorial villosities, or induced pluripotent cells seeded on non-valved patches or valved tubes.^75^–^83^ Even periodontal ligament cells cultured under steady flow environments demonstrated potential for use in heart valve tissue engineering.

Materials made of co-polymer of poly(lactic acid) (PLA) and polycaprolactone (PCL), seeded with human bone-marrow cells, were implanted by Shin’oka et al. in 42 patients with congenital heart diseases in Japan between 2001 and 2005.^84^,^85^ The incidence of early stenosis led this group to go back “from bed to bench” to further understand the mechanisms of this type of early failure.^86^

**Figure 1:**
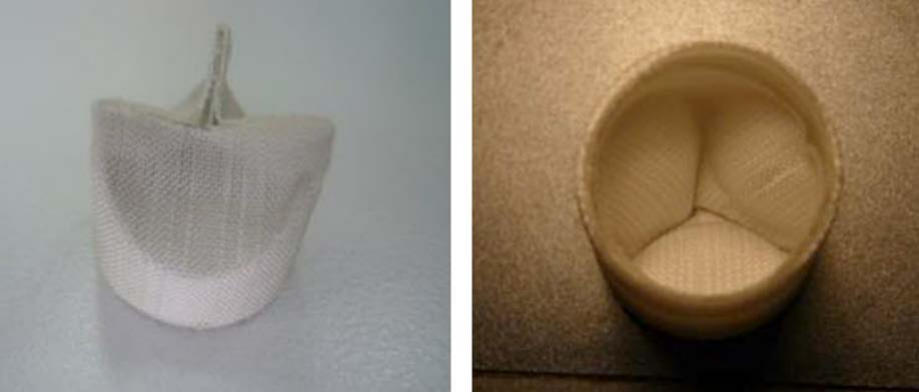
**A Global View of a Bioabsorbable Valve Made of Poly-L-lactic acid (PLLA) and Polyester (PET).** Illustrations from D. Kalfa and P. Menasché’s group.

Prototypes of a bioabsorbable valve and valved tube created using PLLA reinforced with non-absorbable polyester (PET) were assessed as tissue-engineered devices to reconstruct the RVOT by the group of Menasché and Kalfa et al. (Figure 1).

Table 2 summarizes the different types of synthetic polymers used in the research field of the right ventricular outflow tract.

**Table 2:** Different Types of Synthetic Polymers Used in the Research Field of the Right Ventricular Outflow Tract.

**Polymer**	**Cell Type**	**Animal Model**	**Ref.**
Poly(ethylene glycol) (PEG)	Human MSC, valvular interstitial cells (VIC)	n.a.	87–91
Poly(glycolic acid) (PGA)/Poly(lactic acid) (PLA)	Fibroblasts, epithelial cells (EC) and ovine VIC Human fibroblasts, bovine aortic EC	lambs (2 weeks)	92, 93
PGA/Poly-4-hydroxybutyrate (P4HB)	Myofibroblasts, ovine EC Stem cells, endothelial progenitor cells, and ovine valvular endothelial cells (VEC)	lambs (20–100 weeks)	94–98
	
	Human amniotic fluid-derived stem cells	sheep (8 weeks)	
Polycaprolactone (PCL)	Human myofibroblasts	n.a.	99
Poly(glycerol sebacate) (PGS)/PCL	Human umbilical vein-derived endothelial cells (HUVEC)	n.a.	100
Poly(ester urea urethane) (PEUU)	Smooth muscle cells (SMC) from rats	n.a.	101–104
Polydioxaneone (PDO)	Ovine MSC	lambs (1, 4, 8 months)	105
Polycarbonate PCU–Polyhedral oligomeric silsesquioxanes (POSS)	n.a.	n.a.	106

n.a.=not applicable

The concept of decellularization of tissue-engineered heart valves, initially made of bio-degradable synthetic materials and homologous cells, was then introduced to offer an alternative starter matrix for guided tissue regeneration.^107^ This decellularization phase of tissue-engineered heart valves was demonstrated not to alter the collagen structure or tissue strength; it also favored valve performance when compared to their cell-populated counterparts and could provide largely available off-the-shelf homologous scaffolds suitable for reseeding with autologous cells.

Key requirements and properties of those substrates were then discussed in the light of current trends toward designing biologically inspired microenvironments for *in situ* tissue engineering purposes.^108^ The concept of *in situ* tissue engineering, i.e. neotissue regeneration without the use of seeded cells, could solve the disadvantages of using any cell source and achieve a versatile and easier cell-free protocol.^109^ The evaluation of *in situ* tissue engineering vasculature (iTEV) by implantation of scaffolds made of polyglycolide knitted fibers and an L-lactide and ɛ-caprolactone co-polymer sponge in the inferior vena cava of a canine model supported this concept by demonstrating a native tissue-like histological regeneration, with acceptable biomechanical characteristics.^110^

More recently, hundreds of polymers were comprehensively assessed for tissue engineering of cardiac valves, using polymer microarray technology.^111^ Biomechanical tests with real-time displacement and strain mapping were also recently reported to quantify biomechanical and biochemical properties of semilunar heart valve tissues, and potentially facilitate the development of tissue-engineered heart valves.^112^ The role of substrate stiffness in modulating the gene expression and phenotype of neonatal cardiomyocytes *in vitro*^113^ or seeded human bone-marrow stem cells,^114^ on the one hand, and in modulating the activation of valvular interstitial cells,^115^ on the other hand, demonstrated the importance of the mechanical properties of materials used for valve repair or for engineering valve tissue.^116^

Electrospinning appears in the literature as a promising technology to produce scaffolds for cardiovascular tissue engineering. Amoroso et al. evaluated the effect of processing variables and secondary fiber populations on the microstructure and the tensile and bending mechanics of electro-spun biodegradable polyurethane scaffolds for heart valve tissue engineering.^117^ Computational tools were developed in order to describe and predict the mechanical behavior of electrospun valve-shaped scaffolds characterized by different microstructures and showed that a pronounced degree of anisotropy was necessary to reproduce the deformation patterns observed in the native heart valve.^118^

In the emerging field of tissue engineering and regenerative medicine, different design strategies were evaluated to promote the development and evaluation of improved tissue engineering scaffolds. These include mimicking the extracellular matrix, predicting the structural architecture, ensuring adequate initial mechanical integrity, modifying the surface chemistry^109^,^110^,^119^ and topography^120^ to provide cell signaling, and anticipating the material selection so as to predict the required rate of bioresorption.^121^ The biofunctionalization of polymeric scaffolds or decellularized native homografts with motifs (such as RGD, SDF-1α, fibronectin, collagen, CD33) led to encouraging results and could be an alternative way to the complex techniques of cell culture and cell seeding.^109^,^110^,^122^ Prokoph et al. demonstrated that sustained delivery of SDF-1α from proangiogenic hydrogels could effectively attract early endothelial progenitor cells (ePCs), offering a powerful means to trigger endogenous mechanisms of cardiac regeneration.^122^

## NEW FIELDS

### Antenatal Corrective Cardiac Surgery

Embryology and fetal physiopathology of congenital cardiac defects support the idea that the natural progression of some malformations could be curtailed, or arrested altogether, by an intrauterine intervention on the developing heart. Moreover, prenatal diagnosis is performed more and more widely and precisely. This led to the idea of corrective interventions in the fetus, now regarded as a new frontier in pediatric cardiac surgery. Three types of cardiac surgical procedures have been performed so far in the fetus: aortic valvuloplasty in hypoplastic left heart syndrome,^123^,^124^ atrial septostomy to prepare surgery of the same syndrome after birth,^125^ and pulmonary valvuloplasty for pulmonary atresia and hypoplastic right ventricle. Central to progress in this area is the development of instrumentation specifically designed for minimally invasive cardiac surgery in the fetus, involving experts in microengineering and microrobotics. An “ideal” catheter for minimally invasive, fetal cardiac surgery should therefore be appropriately miniaturized and implemented with sensors and driving systems. Some parts of the ideal “fetal catheter” are already available as a prototype.^126^ Such fetal “mechanical” surgical procedures could then be combined with fetal “biological” procedures such as implantation of an appropriate lineage of stem cells or any suitable growth-promoting factor inside the fetal ventricle wall. Collaborations with surgeons, cardiologists, imagers, and engineers will be mandatory to develop such new integrated technologies.

### Robotics

Robotically assisted surgical procedures have been introduced into the field of cardiac surgery since the late 1990s. The da Vinci® Surgical System (Intuitive Surgical, Inc., Sunnyvale, CA) is the only US FDA-approved system for intracardiac procedures. Robotics was first applied in pediatric cardiac surgery for extracardiac procedures such as patent ductus arteriosus ligation and vascular ring divisions.^127^–^129^ Robotically assisted repairs of atrial septal defect were then performed in children.^131^,^132^ There has also been an on-going interest in developing image-guided techniques to perform the same types of intracardiac repairs currently done as open procedures, but without use of cardiopulmonary bypass. To meet this objective, technical advances need to be achieved in two domains: the creation of instruments and devices providing tactile feedback and steerability, on the one hand,^132^ and high-resolution 3D real-time imaging, on the other hand.^133^,^134^ Thus, new catheter-like robotic delivery platforms have been described that facilitate safe navigation and enable complex repairs, such as tissue approximation and fixation, and tissue removal, inside the beating heart.^135^

These new systems combined with enhanced imaging techniques may enable the advancement of the field of beating-heart intracardiac reconstructive interventions currently not feasible with available surgical and catheter-based robotic systems.^136^

## CONCLUSION

These new technologies for structural malformation surgery are still in their infancy but certainly present great promise for the future. Further development of these technologies will depend on the collaboration among diverse medical specialties and the contribution from engineers with special skills. But the translation of these emerging technologies to routine health care and public health policy will also largely depend on economic considerations, value judgments, and political factors.

## Abbreviations

ECM: extracellular matrix;
FDA: Food and Drug Administration;
MSC: marrow stromal cells;
P4HB: poly-4-hydroxybutyrate;
PA: pulmonary artery;
PCL: polycaprolactone;
PCLA: poly-L-lactide;
PGA: polyglycolic acid;
PLA: poly(lactic acid);
PLLA: poly-L-lactic acid;
RV: right ventricle;
RVOT: right ventricular outflow tract;
SIS-ECM: small intestinal submucosa extracellular matrix.

## References

[1] Warnes CA, Williams RG, Bashore TM (2008). ACC/AHA 2008 Guidelines for the Management of Adults with Congenital Heart Disease: a report of the American College of Cardiology/American Heart Association. Task Force on Practice Guidelines (writing committee to develop guidelines on the management of adults with congenital heart disease). Circulation.

[2] Robotin-Johnson MC, Swanson PE, Johnson DC, Schuessler RB, Cox JL (1998). An experimental model of small intestinal submucosa as a growing vascular graft. J Thorac Cardiovasc Surg.

[3] Ruiz CE, Iemura M, Medie S (2005). Transcatheter placement of a low-profile biodegradable pulmonary valve made of small intestinal submucosa: a long-term study in a swine model. J Thorac Cardiovasc Surg.

[4] Wainwright JM, Hashizume R, Fujimoto KL (2012). Right ventricular outflow tract repair with a cardiac biologic scaffold. Cells Tissues Organs.

[5] Scholl FB, Boucek MM, Chan K-C, Valdes-Cruz L, Perryman R (2010). Preliminary experience with cardiac reconstruction using decellularized porcine extracellular matrix scaffold: human applications in congenital heart disease. World J Pediatr Congenital Heart Surg.

[6] Quarti A, Nardone S, Colaneri M, Santoro G, Pozzi M (2011). Preliminary experience in the use of an extracellular matrix to repair congenital heart diseases. Interact Cardiovasc Thorac Surg.

[7] Witt RG, Raff G, Van Gundy J, Rodgers-Ohlau M, Si MS (2013). Short-term experience of porcine small intestinal submucosa patches in paediatric cardiovascular surgery. Eur J Cardiothorac Surg.

[8] Badylak S, Kokini K, Tullius B, Simmons-Byrd A, Morff R (2002). Morphologic study of small intestinal submucosa as a body wall repair device. J Surg Res.

[9] Frigiola A, Redington AN, Cullen S, Vogel M (2004). Pulmonary regurgitation is an important determinant of right ventricular contractile dysfunction in patients with surgically repaired tetralogy of Fallot. Circulation.

[10] Bonhoeffer P, Boudjemline Y, Saliba Z (2000). Percutaneous replacement of pulmonary valve in a right-ventricle to pulmonary-artery prosthetic conduit with valve dysfunction. Lancet.

[11] Bonhoeffer P, Boudjemline Y, Qureshi SA (2002). Percutaneous insertion of the pulmonary valve. J Am Coll Cardiol.

[12] Lurz P, Gaudin R, Taylor AM, Bonhoeffer P (2009). Percutaneous pulmonary valve implantation. Semin Thorac Cardiovasc Surg Pediatr Card Surg Annu.

[13] Lurz P, Coats L, Khambadkone S (2008). Percutaneous pulmonary valve implantation: impact of evolving technology and learning curve on clinical outcome. Circulation.

[14] Nordmeyer J, Khambadkone S, Coats L (2007). Risk stratification, systematic classification, and anticipatory management strategies for stent fracture after percutaneous pulmonary valve implantation. Circulation.

[15] Chowdhury SM Hijazi ZM Rhodes J Early echocardiographic changes after percutaneous implantation of the Edwards SAPIEN transcatheter heart valve in the pulmonary position Echocardiography 2013 2 22 [Epub ahead of print] 10.1111/echo.12147 PMC3817561 23432507

[16] Kalfa D, Loundou A, Nouaille de Gorce Y (2012). Pulmonary position cryopreserved homograft in non-Ross patients: how to improve the results?. Eur J Cardiothorac Surg.

[17] Kalfa D, Feier H, Loundou A (2011). Cryopreserved homograft in the Ross procedure: outcomes and prognostic factors. J Heart Valve Dis.

[18] Kalfa D, Macé L, Metras D, Kreitmann B (2011). How to choose the best available homograft to reconstruct the right ventricular outflow tract. J Thorac Cardiovasc Surg.

[19] Kwak JG, Lee JR, Kim WH, Kim YJ (2010). Mid-term results of the Hancock II valve and Carpentier-Edward Perimount valve in the pulmonary portion in congenital heart disease. Heart Lung Circ.

[20] Brown JW, Ruzmetov M, Rodefeld MD, Vijay P, Turrentine MW (2005). Right ventricular outflow tract reconstruction with an allograft conduit in non-ross patients: risk factors for allograft dysfunction and failure. Ann Thorac Surg.

[21] Yan SM, Mishaly D, Shinfeld A, Raanani E (2008). Right ventricular outflow tract reconstruction: valved conduit of choice and clinical outcomes. J Cardiovasc Med (Hagerstown).

[22] Zachariah JP, Pigula FA, Mayer JE, McElhinney DB (2009). Right ventricle to pulmonary artery conduit augmentation compared with replacement in young children. Ann Thorac Surg.

[23] Belli E, Salihoğlu E, Leobon B (2010). The performance of Hancock porcine-valved Dacron conduit for right ventricular outflow tract reconstruction. Ann Thorac Surg.

[24] Urso S, Rega F, Meuris B (2011). The Contegra conduit in the right ventricular outflow tract is an independent risk factor for graft replacement. Eur J Cardiothorac Surg.

[25] Holmes AA, Co S, Human DG, Leblanc JG, Campbell AI (2012). The Contegra conduit: late outcomes in right ventricular outflow tract reconstruction. Ann Pediatr Cardiol.

[26] Kim WH, Min SK, Choi CH (2007). Follow-up of Shelhigh porcine pulmonic valve conduits. Ann Thorac Surg.

[27] Horere J, Vogt M, Stierle U (2009). A comparative study of mechanical and homograft prostheses in the pulmonary position. Ann Thorac Surg.

[28] Stock UA, Sakamoto T, Hatsuoka S (2000). Patch augmentation of the pulmonary artery with bioabsorbable polymers and autologous cell seeding. J Thorac Cardiovasc Surg.

[29] Sakai T, Li RK, Weisel RD (2001). The fate of a tissue-engineered cardiac graft in the right ventricular out-flow tract of the rat. J Thorac Cardiovasc Surg.

[30] Ozawa T, Mickle DA, Weisel RD, Koyama N, Ozawa S, Li RK (2002). Optimal biomaterial for creation of autologous cardiac grafts. Circulation.

[31] Ozawa T, Mickle DA, Weisel RD (2002). Histologic changes of nonbiodegradable and biodegradable bio-materials used to repair right ventricular heart defects in rats. J Thorac Cardiovasc Surg.

[32] Ozawa T, Mickle DA, Weisel RD (2004). Tissue-engineered grafts matured in the right ventricular outflow tract. Cell Transplant.

[33] Shinoka T, Shum-Tim D, Ma PX (1998). Creation of viable pulmonary artery autografts through tissue engineering. J Thorac Cardiovasc Surg.

[34] Hoerstrup SP, Kadner A, Breymann C (2002). Living, autologous pulmonary artery conduits tissue engineered from human umbilical cord cells. Ann Thorac Surg.

[35] Leyh RG, Wilhelmi M, Rebe P, Ciboutari S, Haverich A, Mertsching H (2006). Tissue engineering of viable pulmonary arteries for surgical correction of congenital heart defects. Ann Thorac Surg.

[36] Stock UA, Nagashima M, Khalil PN (2000). Tissue-engineered valved conduits in the pulmonary circulation. J Thorac Cardiovasc Surg.

[37] Steinhoff G, Stock U, Karim N Tissue engineering of pulmonary heart valves on allogenic acellular matrix conduits: in vivo restoration of valve tissue. Circulation.

[38] Leyh RG, Wilhelmi M, Rebe P (2003). In vivo repopulation of xenogeneic and allogeneic acellular valve matrix conduits in the pulmonary circulation. Ann Thorac Surg.

[39] Leyh R, Wilhelmi M, Haverich A, Mertsching H (2003). [A xenogeneic acellularized matrix for heart valve tissue engineering: in vivo study in a sheep model]. Z Kardiol.

[40] Kim WG, Huh JH (2004). Time related histopathologic changes of acellularized xenogenic pulmonary valved conduits. ASAIO J.

[41] Dohmen PM, Lembcke A, Hotz H, Kivelitz D, Konertz WF (2002). Ross operation with a tissue-engineered heart valve. Ann Thorac Surg.

[42] Dohmen PM, Lembcke A, Holinski S, Pruss A, Konertz W (2011). Ten years of clinical results with a tissue-engineered pulmonary valve. Ann Thorac Surg.

[43] da Costa FD, Dohmen PM, Duarte D (2005). Immunological and echocardiographic evaluation of decellularized versus cryopreserved allografts during the Ross operation. Eur J Cardiothorac Surg.

[44] Simon P, Kasimir MT, Seebacher G (2003). Early failure of the tissue engineered porcine heart valve SYNERGRAFT in pediatric patients. Eur J Cardiothorac Surg.

[45] da Costa FD, Costa AC, Prestes R (2010). The early and midterm function of decellularized aortic valve allo-grafts. Ann Thorac Surg.

[46] Sales VL, Engelmayr GC, Johnson JA (2007). Protein precoating of elastomeric tissue-engineering scaffolds increased cellularity, enhanced extracellular matrix protein production, and differentially regulated the phenotypes of circulating endothelial progenitor cells. Circulation.

[47] Hoerstrup SP, Cummings Mrcs I, Lachat M (2006). Functional growth in tissue-engineered living, vascular grafts: follow-up at 100 weeks in a large animal model. Circulation.

[48] Eckert CE, Mikulis BT, Gottlieb D (2011). Three-dimensional quantitative micromorphology of pre and post-implanted engineered heart valve tissues. Ann Biomed Eng.

[49] Del Gaudio C, Grigioni M, Bianco A, De Angelis G (2008). Electrospun bioresorbable heart valve scaffold for tissue engineering. Int J Artif Organs.

[50] Wang Q, McGoron AJ, Bianco R, Kato Y, Pinchuk L, Schoephoerster RT (2010). In-vivo assessment of a novel polymer (SIBS) trileaflet heart valve. J Heart Valve Dis.

[51] Masoumi N, Jean A, Zugates JT, Johnson KL, Engelmayr GC (2013). Laser microfabricated poly(glycerol sebacate) scaffolds for heart valve tissue engineering. J Biomed Mater Res A.

[52] Flanagan TC, Sachweh JS, Frese J (2009). In vivo remodeling and structural characterization of fibrin-based tissue-engineered heart valves in the adult sheep model. Tissue Eng Part A.

[53] Tedder ME, Simionescu A, Chen J, Liao J, Simionescu DT (2011). Assembly and testing of stem cell-seeded layered collagen constructs for heart valve tissue engineering. Tissue Eng Part A.

[54] Mainwright JM, Hashizume R, Fujimoto KL (2012). Right ventricular outflow tract repair with a cardiac biologic scaffold. Cells Tissues Organs.

[55] Hong H, Dong N, Shi J (2009). Fabrication of a novel hybrid heart valve leaflet for tissue engineering: an in vitro study. Artif Organs.

[56] Duan B, Wu L, Yuan X (2007). Hybrid nanofibrous membranes of PLGA/chitosan fabricated via an electrospinning array. J Biomed Mater Res A.

[57] Chiu YN, Norris RA, Mahler G, Recknagel A, Butcher JT (2010). Transforming growth factor β, bone morpho-genetic protein, and vascular endothelial growth factor mediate phenotype maturation and tissue remodeling by embryonic valve progenitor cells: relevance for heart valve tissue engineering. Tissue Eng Part A.

[58] Chiu LL, Radisic M, Vunjak-Novakovic G (2010). Bioactive scaffolds for engineering vascularized cardiac tissues. Macromol Biosci.

[59] Schleicher M, Wendel HP, Fritze O, Stock UA (2009). In vivo tissue engineering of heart valves: evolution of a novel concept. Regen Med.

[60] Siepe M, Akhyari P, Lichtenberg A, Schlensak C, Beyersdorf F (2008). Stem cells used for cardiovascular tissue engineering. Eur J Cardiothorac Surg.

[61] Kadner A, Hoerstrup SP, Zund G (2002). A new source for cardiovascular tissue engineering: human bone marrow stromal cells. Eur J Cardiothorac Surg.

[62] Mendelson K, Aikawa E, Mettler BA (2007). Healing and remodeling of bioengineered pulmonary artery patches implanted in sheep. Cardiovasc Pathol.

[63] Sales VL, Mettler BA, Lopez-Ilasaca M, Johnson JA, Mayer JE (2007). Endothelial progenitor and mesenchymal stem cell-derived cells persist in tissue-engineered patch in vivo: application of green and red fluorescent protein-expressing retroviral vector. Tissue Eng.

[64] Mettler BA, Sales VL, Stucken CL (2008). Stem cell-derived, tissue-engineered pulmonary artery augmentation patches in vivo. Ann Thorac Surg.

[65] Sales VL, Mettler BA, Engelmayr GC (2010). Endothelial progenitor cells as a sole source for ex vivo seeding of tissue-engineered heart valves. Tissue Eng Part A.

[66] Gottlieb D, Kunal T, Emani S (2010). In vivo monitoring of function of autologous engineered pulmonary valve. J Thorac Cardiovasc Surg.

[67] Van Vlimmeren MA, Driessen-Mol A, Oomens CW, Baaijens FP (2013). The potential of prolonged tissue culture to reduce stress generation and retraction in engineered heart valve tissues. Tissue Eng Part C Methods.

[68] Rubbens MP, Driessen-Mol A, Boerboom RA (2009). Quantification of the temporal evolution of collagen orientation in mechanically conditioned engineered cardiovascular tissues. Ann Biomed Eng.

[69] Ramaswamy S, Gottlieb D, Engelmayr GC (2010). The role of organ level conditioning on the promotion of engineered heart valve tissue development in-vitro using mesenchymal stem cells. Biomaterials.

[70] Martinez C, Rath S, Van Gulden S (2013). Periodontal ligament cells cultured under steady-flow environments demonstrate potential for use in heart valve tissue engineering. Tissue Eng Part A.

[71] Ziegelmueller JA, Zaenkert EK, Schams R (2010). Optical monitoring during bioreactor conditioning of tissue-engineered heart valves. ASAIO J.

[72] Vismara R, Soncini M, Talò G (2010). A bioreactor with compliance monitoring for heart valve grafts. Ann Biomed Eng.

[73] Schmidt D, Dijkman PE, Driessen-Mol A (2010). Minimally-invasive implantation of living tissue engineered heart valves: a comprehensive approach from autologous vascular cells to stem cells. J Am Coll Cardiol.

[74] Emmert MY, Weber B, Behr L (2011). Transapical aortic implantation of autologous marrow stromal cell-based tissue-engineered heart valves: first experiences in the systemic circulation. JACC Cardiovasc Interv.

[75] Kadner A, Hoerstrup SP, Tracy J (2002). Human umbilical cord cells: a new cell source for cardiovascular tissue engineering. Ann Thorac Surg.

[76] Breymann C, Schmidt D, Hoerstrup SP (2006). Umbilical cord cells as a source of cardiovascular tissue engineering. Stem Cell Rev.

[77] Schmidt D, Achermann J, Odermatt B (2007). Prenatally fabricated autologous human living heart valves based on amniotic fluid derived progenitor cells as single cell source. Circulation.

[78] Schmidt D, Mol A, Breymann C (2006). Living autologous heart valves engineered from human prenatally harvested progenitors. Circulation.

[79] Sodian R, Schaefermeier P, Abegg-Zips S (2010). Use of human umbilical cord blood-derived progenitor cells for tissue-engineered heart valves. Ann Thorac Surg.

[80] Sha JM, Yan ZY, Cheng GC (2010). In-vitro seeding of human umbilical cord vein endothelial cells on hydroxyapatite for mechanical heart valve applications. J Heart Valve Dis.

[81] Kadner A, Zund G, Maurus C (2004). Human umbilical cord cells for cardiovascular tissue engineering: a comparative study. Eur J Cardiothorac Surg.

[82] Schmidt D, Breymann C, Weber A (2004). Umbilical cord blood derived endothelial progenitor cells for tissue engineering of vascular grafts. Ann Thorac Surg.

[83] Schmidt D, Mol A, Neuenschwander S (2005). Living patches engineered from human umbilical cord derived fibroblasts and endothelial progenitor cells. Eur J Cardiothorac Surg.

[84] Shin’oka T, Matsumura G, Hibino N (2005). Midterm clinical result of tissue-engineered vascular autografts seeded with autologous bone marrow cells. J Thorac Cardiovasc Surg.

[85] Weber B, Schoenauer R, Papadopulos F (2011). Engineering of living autologous human umbilical cord cell-based septal occluder membranes using composite PGA-P4HB matrices. Biomaterials.

[86] Udelsman BV, Maxfield MW, Breuer CK (2013). Tissue engineering of blood vessels in cardiovascular disease: moving towards clinical translation. Heart.

[87] Benton JA, Fairbanks BD, Anseth KS (2009). Characterization of valvular interstitial cell function in three dimensional matrix metalloproteinase degradable PEG hydrogels. Biomaterials.

[88] Benton JA, Kern HB, Anseth KS (2008). Substrate properties influence calcification in valvular interstitial cell culture. J Heart Valve Dis.

[89] Kloxin AM, Benton JA, Anseth KS (2010). In situ elasticity modulation with dynamic substrates to direct cell phenotype. Biomaterials.

[90] Kloxin AM, Tibbitt MW, Anseth KS (2010). Synthesis of photodegradable hydrogels as dynamically tunable cell culture platforms. Nat Protoc.

[91] Kloxin AM, Kasko AM, Salinas CN, Anseth KS (2009). Photodegradable hydrogels for dynamic tuning of physical and chemical properties. Science.

[92] Shinoka T, Breuer CK, Tanel RE (1995). Tissue engineering heart valves: valve leaflet replacement study in a lamb model. Ann Thorac Surg.

[93] Zund G, Breuer CK, Shinoka T (1997). The in vitro construction of a tissue engineered bioprosthetic heart valve. Eur J Cardiothorac Surg.

[94] Hoerstrup SP, Sodian R, Daebritz S (2000). Functional living trileaflet heart valves grown in vitro. Circulation.

[95] Sodian R, Sperling JS, Martin DP (2000). Fabrication of a trileaflet heart valve scaffold from a polyhydroxy-alkanoate biopolyester for use in tissue engineering. Tissue Eng.

[96] Schmidt D, Mol A, Odermatt B (2006). Engineering of biologically active living heart valve leaflets using human umbilical cord-derived progenitor cells. Tissue Eng.

[97] Dvorin EL, Wylie-Sears J, Kaushal S, Martin DP, Bischoff J (2003). Quantitative evaluation of endothelial progenitors and cardiac valve endothelial cells: proliferation and differentiation on poly-glycolic acid/poly-4-hydroxybutyrate scaffold in response to vascular endothelial growth factor and transforming growth factor beta1. Tissue Eng.

[98] Hoerstrup SP, Kadner A, Melnitchouk S (2002). Tissue engineering of functional trileaflet heart valves from human marrow stromal cells. Circulation.

[99] van Lieshout MI, Vaz CM, Rutten MC, Peters GW, Baaijens FP (2006). Electrospinning versus knitting: two scaffolds for tissue engineering of the aortic valve. J Biomater Sci Polym Ed.

[100] Sant S, Hwang CM, Lee SH, Khademhosseini A (2011). Hybrid PGS-PCL microfibrous scaffolds with improved mechanical and biological properties. J Tissue Eng Regen Med.

[101] Courtney T, Sacks MS, Stankus J, Guan J, Wagner WR (2006). Design and analysis of tissue engineering scaffolds that mimic soft tissue mechanical anisotropy. Biomaterials.

[102] Stankus JJ, Guan J, Fujimoto K, Wagner WR (2006). Microintegrating smooth muscle cells into a biodegradable, elastomeric fiber matrix. Biomaterials.

[103] Stella JA, D’Amore A, Wagner WR, Sacks MS (2010). On the biomechanical function of scaffolds for engineering load-bearing soft tissues. Acta Biomater.

[104] Stella JA, Liao J, Hong Y, David Merryman W, Wagner WR, Sacks MS (2008). Tissue-to-cellular level deformation coupling in cell micro-integrated elastomeric scaffolds. Biomaterials.

[105] Kalfa D, Bel A, Chen-Tournoux A (2010). A polydioxa-none electrospun valved patch to replace the right ventricular outflow tract in a growing lamb model. Biomaterials.

[106] Kidane AG, Burriesci G, Edirisinghe M, Ghanbari H, Bonhoeffer P, Seifalian AM (2009). A novel nanocomposite polymer for development of synthetic heart valve leaflets. Acta Biomater.

[107] Dijkman PE, Driessen-Mol A, Frese L, Hoerstrup SP, Baaijens FP (2012). Decellularized homologous tissue-engineered heart valves as off-the-shelf alternatives to xeno- and homografts. Biomaterials.

[108] Bouten CV, Dankers PY, Driessen-Mol A, Pedron S, Brizard AM, Baaijens FP (2011). Substrates for cardiovascular tissue engineering. Adv Drug Deliv Rev.

[109] Patterson JT, Gilliland T, Maxfield MW (2012). Tissue-engineered vascular grafts for use in the treatment of congenital heart disease: from the bench to the clinic and back again. Regen Med.

[110] Matsumura G, Nitta N, Matsuda S (2012). Long-term results of cell-free biodegradable scaffolds for in situ tissue-engineering vasculature: in a canine inferior vena cava model. PLoS One.

[111] Peragallo S, Tura O, Wu M (2012). Novel biopolymers to enhance endothelialisation of intra-vascular devices. Adv Healthc Mater.

[112] Huang HY, Balhouse BN, Huang S (2012). Application of simple biomechanical and biochemical tests to heart valve leaflets: implications for heart valve characterization and tissue engineering. Proc Inst Mech Eng H.

[113] Forte G, Gliari S, Ebara M (2012). Substrate stiffness modulates gene expression and phenotype in neonatal cardiomyocytes in vitro. Tissue Eng Part A.

[114] Emani S, Mayer JE, Emani SM (2011). Gene regulation of extracellular matrix remodeling in human bone marrow stem cell-seeded tissue-engineered grafts. Tissue Eng Part A.

[115] Quinlan AM, Billiar KL (2012). Investigating the role of substrate stiffness in the persistence of valvular inter-stitial cell activation. J Biomed Mater Res A.

[116] van Vlimmeren MA, Driessen-Mol A, Oomens CW, Baaijens FP (2012). Passive and active contributions to generated force and retraction in heart valve tissue engineering. Biomech Model Mechanobiol.

[117] Amoroso NJ, D’Amore A, Hong Y, Rivera CP, Sacks MS, Wagner WR (2012). Microstructural manipulation of electrospun scaffolds for specific bending stiffness for heart valve tissue engineering. Acta Biomater.

[118] Argento G, Simonet M, Oomens CW, Baaijens FP (2012). Multi-scale mechanical characterization of scaffolds for heart valve tissue engineering. J Biomech.

[119] Baumann L, Prokoph S, Gabriel C, Freudenberg U, Werner C, Beck-Sickinger AG (2012). A novel, biased-like SDF-1 derivative acts synergistically with starPEG-based heparin hydrogels and improves eEPC migration in vitro. J Control Release.

[120] Nikkah M, Edalat F, Manoucheri S, Khademhosseini A (2012). Engineering microscale topographies to control the cell-substrate interface. Biomaterials.

[121] Chung S, King MW (2011). Design concepts and strategies for tissue engineering scaffolds. Biotechnol Appl Biochem.

[122] Prokoph S, Chavakis E, Levental KR (2012). Sustained delivery of SDF-1α from heparin-based hydrogels to attract circulating pro-angiogenic cells. Biomaterials.

[123] Bacha EA (2011). Impact of fetal cardiac intervention on congenital heart surgery. Semin Thorac Cardiovasc Surg Pediatr Card Surg Annu.

[124] Kohl T, Sharland G, Allan LD (2000). World experience of percutaneous ultrasound-guided balloon valvuloplasty in human fetuses with severe aortic valve obstruction. Am J Cardiol.

[125] Marshall AC, van der Velde ME, Tworetzky W (2004). Creation of an atrial septal defect in utero for fetuses with hypoplastic left heart syndrome and intact or highly restrictive atrial septum. Circulation.

[126] Coceani F, Menciassi A, Murzi B (2010). Antenatal corrective cardiac surgery: an emerging area for technological innovation. Minim Invasive Ther Allied Technol.

[127] Le Bret E, Papadatos S, Folliguet T (2002). Interruption of patent ductus arteriosus in children: robotically assisted versus videothoracoscopic surgery. J Thorac Cardiovasc Surg.

[128] Mihaljevic T, Cannon JW, del Nido PJ (2003). Robotically assisted division of a vascular ring in children. J Thorac Cardiovasc Surg.

[129] Suematsu Y, Mora BN, Mihaljevic T, del Nido PJ (2005). Totally endoscopic robotic-assisted repair of patent ductus arteriosus and vascular ring in children. Ann Thorac Surg.

[130] Bacha EA, Bolotin G, Consilio K, Raman J, Ruschhaupt DG (2005). Robotically assisted repair of sinus venosus defect. J Thorac Cardiovasc Surg.

[131] Argenziano M, Oz MC, Kohmoto T (2003). Totally endoscopic atrial septal defect repair with robotic assistance. Circulation.

[132] Butler E, Folk C, Cohen A (2011). Metal MEMS tools for beating-heart tissue approximation. IEEE Int Conf Robot Autom.

[133] Vasilyev NV, Melnychenko I, Kitahori K (2008). Beating-heart patch closure of muscular ventricular septal defects under real-time three-dimensional echocardiographic guidance: a preclinical study. J Thorac Cardiovasc Surg.

[134] Deng J, Rodeck CH (2006). Current applications of fetal cardiac imaging technology. Curr Opin Obstet Gynecol.

[135] Vasilyev NV, Dupont PE, del Nido PJ (2012). Robotics and imaging in congenital heart surgery. Future Cardiol.

[136] Cannon JW, Howe RD, Dupont PE, Triedman JK, Marx GR, del Nido PJ (2003). Application of robotics in congenital cardiac surgery. Semin Thorac Cardiovasc Surg Pediatr Card Surg Annu.

